# Genetic diversity and relationship of Dulong chickens using mitochondrial DNA control region

**DOI:** 10.1080/23802359.2019.1700837

**Published:** 2019-12-13

**Authors:** Qingqing Li, Pu Zhang, Momo Li, Lu Li, Yaodong Hu, Shailendra Kumar Mishra, Aiwei Guo, Guangyu Li, Diyan Li, Yanqing Duan

**Affiliations:** a Key Laboratory for Forest Resources Conservation and Utilization in the Southwest Mountains of China, Ministry of Education, Southwest Forestry University, Kunming, Yunnan, China;; b Life Science College, Southwest Forestry University, Kunming, Yunnan, China;; c Kunming Xianghao Technology Co. Ltd., Kunming, Yunnan, China;; d Farm Animal Genetic Resources Exploration and Innovation Key Laboratory of Sichuan Province, Sichuan Agricultural University, Chengdu, Sichuan, China;; e School of Mathematics and Computer Science, Yunnan Nationalities University, Kunming, Yunnan, China;; f Technology Center, China Tobacco Yunnan Industrial Co., Ltd., Kunming, Yunnan, China

**Keywords:** Genetic diversity, Dulong chicken, mitochondrial DNA

## Abstract

The genetic structure and evolutionary relationship of Dulong chicken with other native Chinese species remained unclear. In this study, the mitochondrial control region was analyzed in total of 343 samples comprising 59 from Dulong chicken and 284 from 8 other Chinese local breeds revealed 51 mutation sites that defined 42 haplotypes. The maximum genetic variation was observed between the Shimian caoke and Pengxian yellow chickens. Phylogenetic analysis revealed that these local chickens mainly scatter in two southwestern clades. Dulong chickens have close relationship with other native chicken. Finding of this study suggests a single matrilineal lineage of indigenous Dulong chickens.

## Introduction

Dulong Chicken is an ancient unique local breed, raised by a very few ethnic Dulong people and distributed only in the alpine valley of Gongshan County, Nujiang, Yunnan province of China. In 2010, it was listed in the national catalog of livestock and poultry genetic resources (Chen 2013). Dulong chickens contribute substantially to meat and egg production with rough feeding and low production costs. More importantly, Dulong chicken has strong disease resistance (Huang et al. 2007). These unique traits have long been domesticated under relatively closed geographical conditions and are important genetic material and storage for improved chicken breed reproduction. At the same time, due to the relatively harsh growing environment and semi-wild state, the Dulong chicken is adapted to the climate conditions of high altitude and high humidity and has strong adaptability to the local ecological environment.

The genetic attributes and evolution mechanism of Dulong chicken have not been well studied. Microsatellite based study suggested that the Dulong chicken had abundant polymorphisms and unique alleles that were not found in other chicken breeds (Huang et al. 2007), demonstrating that there was no communication with other chicken breeds in the evolutionary history of the Dulong chicken. Zhou et al. (2008) discussed the systematic status of 18 Dulong chickens using microsatellite DNA markers. They speculated that the Dulong chicken had been evolved from red jungle fowl as it falls with Tibetan chickens and Chahua chickens in same cluster. They concluded that the genetic structure of Dulong chicken was relatively closer to the chickens in south and southwest China, while distant from the chickens in north and northeast China, which was same as that of the red jungle fowl. It is obvious that the existing research on the genetic information data acquisition and in-depth analysis of Dulong chicken is very limited, so it is necessary to explore the genetic diversity of the Dulong chicken population using more genetic markers.

Mitochondrial DNA (mtDNA) has proven to be particularly useful in the study of phylogeny and relatedness of population owing to its solely matrilineal inheritance (Giles et al. 1980), compact organization (Melnick and Hoelzer 1993), hence absence of recombination, and high nucleotide substitution rate (Brown et al. 1992). D-loop of mitochondria has a non-coding region involved in mitochondrial genome replication and considered target site is hypervariable. The rapid rate of evolution resulting high level of diversity makes mtDNA particularly useful for detecting population genetic structure at the intraspecific level. The even higher rate of nucleotide substitution in the control region of mtDNA makes this region especially informative in studies of human evolution, forensic identity testing, and population genetics (Parsons et al. 1997).

In this study, we analyzed partial mtDNA control region sequences of semi-wild Dulong chickens and 8 chicken breeds sampled throughout Sichuan province in China, to determine whether genetic attributes support the existence of regional geographic differences of Dulong chicken. Our findings will enhance the understanding of matrilineal relationship of Dulong chicken with other local populations.

## Materials and methods

### Sampling and DNA extraction

In total, 59 and 284 blood samples were collected from Yunnan (Dulong chicken) and 8 Sichuan chicken breeds (27 Pengxian yellow chickens, 29 Jinyang silky chickens, 30 Emei Black chickens, 40 Jiuyuan black chickens, 29 Muchuan silky chickens, 39 Miyi chickens, 46 Shimian Caoke chickens, and 44 Tianfu silky chickens). All blood samples were stored at −20°0 to ensure the completeness of mitochondria DNA. And all experimental procedures were approved by the Institutional Animal Care and Use Committee of the Institute of Animal Genetic and Breeding, Sichuan Agricultural University, Chengdu, China, under permit No. DKY-B20111210. Protienase-K/Phenol-chloroform standard procedure was used to extract genomic DNA from blood samples as previously described (Sambrook et al. 1989).

The partial mtDNA control region (about 500 bp) was PCR-amplified using previously reported primers: F: 5′-AGGACTACGGCTTGAAAAGC-3′ (Fumihito et al. 1994) and R: 5′-ATGTGCCTGACCGAGGAACCAG-3′ (Liu et al. 2006). PCR amplification was carried out in a 25-μL volume containing 12.5 μL of 2 × Taq PCR Master Mix, 1.25 μL of template genomic DNA, 1.25 μL of each primer (10 pmol/μL), and 8.75 μL of ddH_2_O. After incubation at 95 °C for 3 min, 33 cycles were performed as follows: 94 °C for 35 s, 58.4 °C for 35 s and 72 °C for 1 min, with a final extension at 72 °C for 10 min. The sequencing was conducted using ABI 3,730 automated sequencer (Applied Biosystems, Carlsbad, CA). Nucleotide Basic Local Alignment Search Tool (BLASTN) was used to ensure that the sequenced fragment of DNA was target sequence. The obtained sequences of the partial control region were annotated manually using Chromas vs2.0 (http://www.technelysium.com.au/chromas.html). The sequences were aligned using ClustalX 2.1 program in the DNASTAR package (DNASTAR Inc., Madison, WI) (Larkin et al. 2007) and truncated to 482 bp corresponding to longest sequence common to all samples for further analysis. Nucleotide diversity, average number of nucleotide differences, haplotypes and Tajima’s D values were calculated using DnaSP vs6.0 (Rozas et al. 2017). The evolutionary relationships among unique sequences were estimated using the neighbor-joining model with the *p*-distance method using software MEGA7 (Kumar et al. 2016). The maximum parsimony median-joining (MJ) network (Bandelt et al. 1999) drown for the 334 sequences of control region by using NETWORK program (fluxus-engineering.com).

## Results

### Genetic diversity of 9 chicken breeds

The mtDNA of 343 individuals from Yunnan and Sichuan local breeds were classified into 42 unique haplotypes (Hap1–Hap42) by 51 substitutions (10.58%) of 482 nucleotide positions, consisting of 6 transversions and 45 transitions. Out of 42 unique haplotypes, fourteen singletons included the highest of 4 in Dulong chicken, followed by 3 in Emei black and Miyi fowl, 2 in Jinyang silky fowl and 1 in Pengxian yellow & Jiuyuan black-bone fowl when analyzed across the breed. Four major haplotypes viz. Hap3, Hap4, Hap7, and Hap10 were found across all nine breeds comprising, 69, 74, 22, and 28 representative individuals, respectively.

Among 51 variable sites, 41 were Parsimony-informative sitesand 10 were singleton variable sites. The overall nucleotide polymorphism information of Dulong chickens, as well as other breeds, are summarized in Table 1. The number of polymorphic sites among each breed varied from lowest range of 12 in Muchuan to highest 34 in Dulong. The Muchuan chicken had lowest (5) while Dulong chicken had highest (16) unique sequences. Among the nine populations, the highest haplotype diversity (0.911), nucleotide diversity (0.016) and the average number of nucleotide differences (7.730) were found in Dulong chickens (Table 1). Pengxian chicken was found to have lowest range of haplotype diversity. The estimated Tajima’s D selective neutrality statistics were not significant among all chicken breeds, suggested that no demographic expansion among the breeds. Among them, Tianfu black-bone fowl and Jiuyuan black-bone fowl showed negative Tajima’s D values indicated these species are under purifying selection.

**Table 1.** Nucleotide polymorphism of mtDNA control region within 9 chicken breeds.

	Variable sites	Parsim-info sites	Singleton sites	Haplotypes	Haplotype diversity	Nucleotide diversity	Average No. of nucleotide differences	Tajima’s D*
Emei	17	9	8	10	0.786	0.010	4.784	0.392
Jinyang	22	17	5	8	0.700	0.012	5.813	0.133
Jiuyuan	18	11	7	7	0.709	0.007	3.492	−0.570
Miyi	26	24	2	15	0.906	0.015	7.387	0.686
Muchuan	12	11	1	5	0.732	0.007	3.167	0.120
Pengxian	20	12	8	7	0.641	0.011	5.202	0.009
Shimian	19	19	0	7	0.741	0.011	4.817	0.367
Tianfu	20	17	3	10	0.844	0.009	4.539	−0.414
Dulong	34	29	4	16	0.911	0.016	7.730	0.086
Total	51	41	10	42	0.893	0.014	6.791	−0.509

Dulong: Dulong chicken; Pengxian: Pengxian yellow chicken; Jinyang: Jinyang silky chicken; Emei: Emei Black chicken; Jiuyuan: Jiuyuan black chicken; Muchuan: Muchuan silky chicken; Miyi: Miyi chicken; Shimian: Shimian Caoke chicken; Tianfu: Tianfu silky chicken. *All of them were not statistically significant in this study (*p*-values > 0.1).

**Figure 1.**
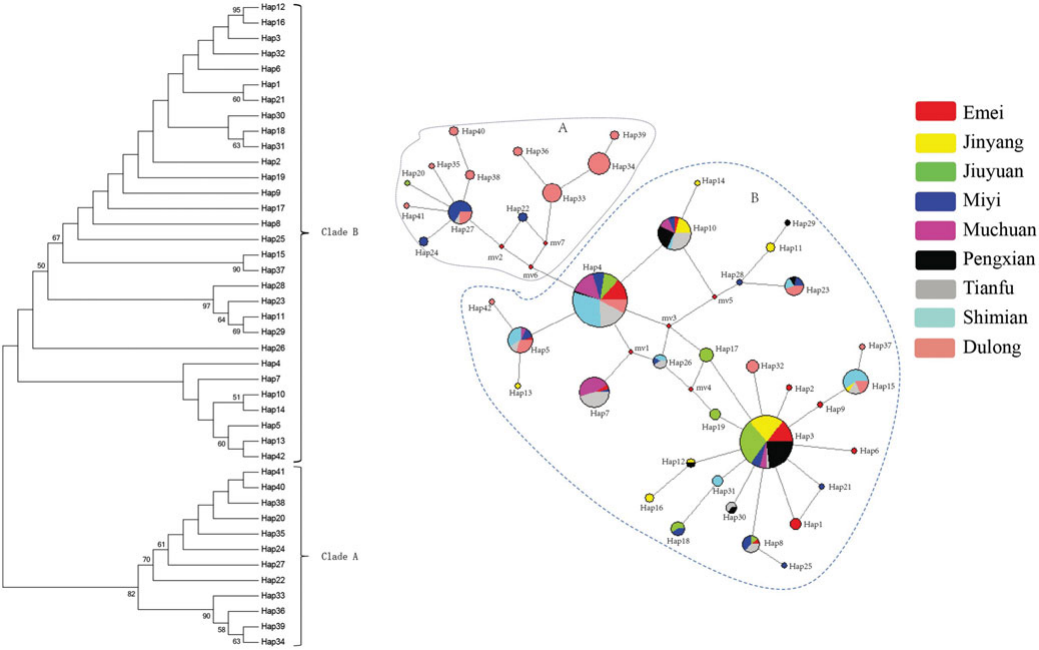
Evolutionary relationship of all chicken samples. *Note*. the image on the left: evolutionary relationship of 42 unique partial control region sequences from 343 chickens constructed using the neighbor-joining model with the *p*-distance method. The reliability of the estimated trees was evaluated by the bootstrap method with 1000 replications. Only bootstrap values more than 50% are shown in this figure. The image on the right: maximum parsimony median-joining network of Dulong chickens with other Sichuan local chicken breeds based on 482 bp control region sequence. Node sizes are proportional to haplotype frequencies. The lines linking the nodes are proportional to the mutation steps. Red nodes (mv1–mv7) indicate inferred steps not identified in the sampled populations. Colors within the circles represent chickens different localities at which each haplotype was detected.

There are 10 haplotypes (Hap32–Hap42) that were specifically existed in Dulong chicken (Figure 1). Dulong chickens have the highest number of haplotypes (16) mostly appeared as singleton followed by Miyi chickens that have 15 haplotypes. Muchuan and Tianfu silky fowls have five and ten haplotypes, respectively, and all were shared with other chicken breeds. Dulong chicken shared four haplotypes with Tianfu, Shimian, and Miyi chickens. On the other hand, the Dulong chicken did not share the haplotype with Jiuyuan, and only one with Jinyang chicken. All the unique haplotypes of Emei black chickens appeared around the major haplotype 3. Further Sichuan local chickens shared haplotypes extensively with each other and showed their presence in all the four major expanding haplotypes, however, only few haplotypes were found unique for each breed.

### Nucleotide divergence and genetic differentiation between populations

Further analysis using the mitochondrial control region, the average number of nucleotide substitutions per site (*Dxy*), and net divergence (*Da*) were calculated between populations according to Nei (1987) and Tajima (1983) and are shown in Table 2, we estimated that the *Dxy* between the nine chicken breeds was ranged from lowest in the Tianfu 0.802 to highest 2.094 in Dulong. The *Da* was measured maximum 0.881 between Dulong and other chicken populations while the divergence was almost negligible between Muchuan and Tianfu silky fowls (−0.003).

**Table 2.** Coefficient of differentiation (*Gst*), gene flow (*Nst*), Nucleotide divergence (*Dxy*) and net genetic distance (*Da*) between each pair of chicken breeds.

	Emei	Jinyang	Jiuyuan	Miyi	Muchuan	Pengxian	Shimian	Tianfu	Dulong
Coefficient of differentiation and gene flow									
Emei		1.841	7.071	14.482	28.150	0.684	16.239	19.577	29.791
Jinyang	4.972		3.823	17.933	35.924	−2.701	22.760	26.973	32.638
Jiuyuan	1.753	2.539		27.880	51.426	3.800	37.464	41.867	43.063
Miyi	3.917	7.440	6.564		18.811	17.858	8.399	11.389	12.033
Muchuan	3.349	11.815	8.952	5.732		35.885	10.451	−0.340	26.607
Pengxian	5.285	−0.939	2.851	8.370	12.470		22.916	26.639	33.130
Shimian	5.264	15.898	12.530	6.804	5.601	16.865		3.351	17.236
Tianfu	3.955	9.767	9.446	3.233	0.485	10.340	3.959		20.747
Dulong	5.684	9.322	9.118	2.780	6.772	10.339	6.370	4.575	
Nucleotide divergence and net genetic distance									
Emei		0.021	0.066	0.214	0.324	0.007	0.195	0.236	0.551
Jinyang	1.127		0.039	0.300	0.524	−0.030	0.328	0.398	0.680
Jiuyuan	0.930	1.010		0.437	0.733	0.036	0.520	0.602	0.881
Miyi	1.485	1.678	1.573		0.256	0.285	0.119	0.161	0.216
Muchuan	1.154	1.462	1.428	1.358		0.488	0.097	−0.003	0.413
Pengxian	1.050	1.120	0.943	1.599	1.362		0.312	0.369	0.665
Shimian	1.197	1.438	1.388	1.393	0.931	1.358		0.034	0.274
Tianfu	1.209	1.479	1.441	1.406	0.802	1.386	1.011		0.336
Dulong	1.857	2.094	2.052	1.794	1.551	2.015	1.583	1.616	

The upper half part: the *Gst* (below diagonal) and the *Nst* (above diagonal), all the values were enlarged 100 times. The maximum and minimum values are underlined. The lower half part: the *Dxy* (lower diagonal) and the *Da* (upper diagonal), all the values were enlarged 100 times. The maximum and minimum values are underlined.

We attempted to estimate the Nei’s (1973) *Gst* and *Nst* as pairwise measures of genetic subdivision between these local chicken populations (Table 2). The population differentiation, *Gst* values were calculated for all the possible population pairs. The maximum differentiation was found between the Shimian caoke chickens and Pengxian yellow chickens while the minimum differentiation was found between the Jinyang silk fowls and Pengxian yellow fowls. Furthermore, Dulong chickens showed the greatest genetic distance with Jiuyuan (*Nst* = 43.063) and closer to Miyi fowls (*Nst* = 12.033).

### Phylogenetic relationships among Dulong chicken and indigenous chickens

The neighbor-joining tree was generated using 42 unique sequences to estimate evolutionary genetic distances by the maximum composite likelihood method using MEGA7 program. The phylogenetic analysis showed the clustering of these haplotypes into two maternal lineages (Figure 1). The analysis revealed genetic continuity among chickens of Sichuan basin region of China which formed a separate lineage from the Chickens of the Dulong county area.

The Network analysis of the 42 unique chicken partial control region sequences depicts the minimum mutational distances among the haplotypes and is consistent with features of the neighbor-joining tree (Figure 1). Haplotypes within haplogroups A and B were differed by at least 6 nucleotide substitutions. The haplotypes in the intra haplogroup were closer to each other than inter haplogroup.

## Discussion

The population size of Dulong chicken is dwindling rapidly, however, the specific inherited characteristics of Dulong chickens are far from comprehensive, and their potential value has yet to be discovered (Li et al. 2017). Thus, tapping the genetic information of this endangered semi-wild chicken breed requires a timely and effective approach, especially considering the value and importance of this local genetic resource. The mitochondrial control region is highly polymorphic as reported by several researchers worldwide (Liu et al. 2006; Li et al. 2011; Zhang et al. 2017).

In the present study, we used mtDNA partial control region sequences to investigate the genetic diversity and genetic differentiation of Dulong chicken and 8 other indigenous fowls. Our findings will increase the understanding of the genetic diversity of the Dulong chickens in China. All of the genetic diversity parameters in Dulong chickens showed differences from other lowland chickens of different localities, suggesting that genetically Dulong chickens are different from the other indigenous chickens of Sichuan basin. Interestingly, we found that Dulong chicken and Miyi chicken habitat in high altitude and showed higher genetic diversity than the other chicken breeds. Thus, further study should be conducted to explore whether there is a relationship between altitude and genetic diversity.

A high genetic difference were observed between these nine chicken breeds which showed a pattern similar to that obtained in the previous investigation of Chinese local chicken populations (Liu et al. 2006) The value of average number of nucleotide differences is higher (0.016) in Dulong chicken than other Sichuan local chicken breeds (0.007–0.015) suggested that those sequences are potentially isolated from other chickens. Compared with genetic diversity study of *COX-III* gene in lowland (Muchuan, Emei, Jiuyuan, and Tianfu) and Tibetan chickens (Liu et al. 2017), and the study of nuclear gene bitter taste receptor gene family in Sichuan domestic and Tibetan chicken populations (Su et al. 2016), the control region showed extensive genetic diversity (average number of nucleotide difference (*K*) ranged from 3.167 to 7.730) than *COX-III* gene (*K* ranged from 0.720 to 1.621) and *Tas2rs* (0.344–3.564). A high level of diversity makes control region particularly useful for detecting population genetic structure at the intraspecific level. Our results revealed that there are more shared haplotypes among Sichuan local chickens while introgression of the Dulong chicken breed is limited, which indicated this breed was only raised in Dulong county area.

Demographic analyses (Tajima’s D) using partial control region sequence polymorphism showed a signal of population expansion in Tianfu black-bone fowl and Jiuyuan black-bone fowl, and a signal of population contraction in Dulong and other 6 chicken breeds. But Tajima’s D values were not significant in all chicken breeds, indicating that neither balancing selection nor purifying selection occurred in all chicken breeds. Previous studies also found that Tajima’s D values were not significant in all Chinese chicken populations (Zhang et al. 2017) and in four varieties of Thai indigenous chickens (*p* > 0.05) (Teinlek et al. 2018). On the other hand, a study of five Tanzanian chicken ecotypes showed that Unguja tested a significant positive value, while Kuchi recorded a significant negative value (*p* < 0.05) (Lyimo et al. 2013). A study of maternal origin of Turkish and Iranian native chickens inferred from mitochondrial DNA D-loop sequences showed that White Marandi (−2.102, *p* < 0.05) and common breed (−2.092, *p* < 0.05) chickens have significantly negative Tajima’s D values (Meydan et al. 2016) that indicated a population expansion in these chicken populations. Although the Tajima’s D values were not significant in these Chinese local chicken populations, most of them were experiencing a decline in their numbers. It is necessary to take appropriate measures to protect animal genetic resources of these domestic chicken breeds, especially make full use of their good disease resistance characteristics.

The values of *Dxy* exhibit the extent of differences between the breeds on the basis of nucleotide substitution, the more the differences the more the divergence between the breeds. The results indicated that there is little difference among the Sichuan local chicken populations while the majority of differences occur between the Dulong chickens and Jiyang silk fowls. Miyi fowls showed the closest net genetic distance to Dulong Chicken. It might be because Miyi and Dulong are geographically closest. In this study, we found that haplogroup B is the largest, most geographically dispersed, it is the most likely to be the basal haplogroup. A previous study showed that 39.696 and 35.358% individuals of Sichuan province take proportion of 47.059 and 15.686% haplotypes in their Clade A and Clade E, respectively (Zhang et al. 2017). However, haplotypes of haplogroup B correspondence to previous Clade A or Clade E need further study to explore.

In summary, we sequenced and compared Dulong chickens with 8 different chicken breeds in this study. The results indicated that the Dulong chickens exhibit more genetic diversity, and they may have little gene communication with other chickens. Additionally, the result of evolutionary relationships and M–J Network provides evidence of hybridization is arising between Dulong and other chicken breeds. The obtained mtDNA data extend our understanding of the genetic diversity of Dulong chickens and more genetics or genomics studies need to be conducted in order to reveal the clear gene flow between or among different breeds in the future.

## Data Availability

The mitochondrial DNA control region data were submitted to NCBI with the GenBank accession numbers MN381367 and MN381709.
